# Arsenic in Mennorrhagia, Leucorrhæa, &c.

**Published:** 1860-05

**Authors:** Arthur P. Burns

**Affiliations:** Ellicots Mills, Md.


*Arsenic in Mennorrhagia, Leucorrhœa, &c.* By Arthur P. Burns, M. D., of Ellicots Mills, Md.

He said, my usual plan of treatment has been, in Menorrhagia, if called to the patient during hemorrhage, to give immediately ten to twenty drops of Fowler's solution according to the severity of the case, and repeat it in doses of ten drops, every fifteen to twenty minutes, till the hemorrhage is checked. I have never had occasion to push it to a dangerous amount. Care must be exercised in its administration, as it will entirely suspend the menstrual secretion. I then give five to ten drops three times a day, during the menstrual period, and in the interval, three to five drops, three times a day, and steadily persevere in the use of it until a cure is effected. Sometimes I use injections, counter irritations to the sacrum by blisters, &c. In either affection if there is debility I use tinct. cinchona comp. ℥ iii, tinct. cantharidis ʒ ii. Mix, dose a teaspoonful three times a day. I sometimes add spts. ether nitr., and tinct. opii. camphor.

I may state here that I have persevered for months continuously in the use of the remedy, without any unpleasant effects. I know of no remedy so effective and so prompt in arresting hemorrhage in threatened abortion. It seems to suspend at once the contractions as well as the hemorrhage. I usually give twenty drops for the first dose, and ten drops every fifteen to twenty minutes thereafter, until the hemorrhage is checked. In hemorrhage after delivery, it is equally efficacious used in the same manner and doses in excessive or long continued lochial discharges in doses of five to ten drops three times a day. In conjunction with the tonic mixture, it acts promptly and efficaciously. I have had cases that resisted other treatment yield speedily to this.

In one case in which the discharge continued for weeks after the usual time, with occasional hemorrhages resisting other treatment it yielded promptly to ten drops *ter die*, and the tonic mixture. Its modus operandi I am unacquainted with, that it does not act by inducing uterine contractions appears certain, as it will suspend them in threatened abortion, and I believe, in large doses would suspend them at the full period of gestation. I believe it to be a Hemostatic of great power, though I have never had a fair opportunity of testing its powers, and equally as efficacious in Hæmoptysis, &c., as it is in Menorrhagia. A. J. M. L.

Since reading the above article, we have had an opportunity to test the merits of Arsenic in several cases of Menorrhagia. In all of them it proved successful, without producing any unpleasant effects. We have used it in Dysmenorrhæa, and Leucorrhæa, with results more satisfactory than anything previously used. We suggested its use to a neighboring physician, in a very alarming case of uterine hemorrhage from placenta previa, some five weeks before accouchment, where there had been previous attacks of a very threatened character, and at all subsequent attacks it controlled hemorrhage, but always produced a strong and persistent uterine aching; the woman went her full period, and had a speedy and safe delivery under the care of Dr. Campbell, with no unusual hemorrhage, or untoward symptoms.

From the number of above named cases in which we have used arsenic, we think it a valuable remedy and deserving the attention of the profession. Yet we cannot go so far as Dr. Burns and say we believe it would suspend uterine contraction at the full period of gestation if given in large doses, unless the dose was large enough to suspend respiration and circulation. Suspending uterine contraction in threatened abortion, and at the full period of gestation is a different action altogether. The former is a pathological condition and the latter a physiological or functional one. The therapeutical effect, or modus operandi of medicine, in the two conditions is very different. Opium will suspend uterine contractions in threatened abortion, yet in normal labor, will have but little effect, unless in very large doses.—Ed.

[*Belmont Medical Journal.*

